# Implementing collaborative practices in healthcare settings using champions: a scoping review

**DOI:** 10.1186/s13012-025-01463-2

**Published:** 2025-11-04

**Authors:** Robin Lüchinger, Marie-Claude Audétat, Katherine Blondon, Noëlle Junod Perron

**Affiliations:** 1 Unit of Development and Research in Medical Education, Faculty of Medicine, University of Geneva, Rue Michel-Servet 1, Genève 4, 1211 Switzerland; 2 University Institute of Family and Child Medicine, Faculty of Medicine, University of Geneva, Geneva, Switzerland; 3 Medical Directorate, University Hospitals of Geneva, Geneva, Switzerland; 4 Interprofessional Simulation Center, Faculty of Medicine, University of Geneva, Geneva, Switzerland

**Keywords:** Implementation research, Champions, Scoping review, Collaboration, Healthcare, Collaborative practice, Barriers, Facilitators

## Abstract

**Objective:**

The aim of this scoping review was to investigate the published literature on the use of champions to implement collaborative practices in healthcare.

**Methods:**

A systematic review of the literature was conducted, and PubMed and Embase were screened for the period of 01.2000—02.2025. Two pairs of researchers conducted the articles selection. Three researchers extracted the data, and two researchers analyzed the data regarding the type of projects involving local champions, their focus in terms of collaborative practices and as well as the facilitators and barriers local champions face in implementing collaborative practice.

**Results:**

From 1768 articles, 41 were included and underwent full data extraction. Most articles were monocentric, qualitative and half of them had a clear focus on the effects of championing to implement collaborative practices. Champions covered a variety of professions and were mainly integrated in multi-professional implementation teams. Descriptions of champions’ characteristics, training, roles and responsibilities were sparse and vague. Collaborative practices were regularly integrated in a broader project and were not the implementation’s focus. Conceptual frameworks to guide implementation efforts were used in less than a third of the included articles. Most enabling and disabling factors were related to the internal organizational context. Factors such as innovation’s fit or partnerships and collaborations influencing the implementation were mostly reported as change facilitators.

**Conclusion:**

Our results highlight that although champions are recognized for their ability to drive change, little is known about how they are selected, trained, and positioned within institutions and how effective they are in implementing changes in collaborative practices. Use of a theoretical framework to guide the implementation process may help define outcome measures as well as better clarify the factors facilitating or impending such process.

**Supplementary Information:**

The online version contains supplementary material available at 10.1186/s13012-025-01463-2.

Contribution to the literature

- Research has demonstrated the need to standardize the presentation of results from implantation processes by providing guidelines and recommendations. The use of frameworks linked to implementation science must continue to be strengthened to guarantee the generalizability and reproducibility of results.
- The recommendations concerning the use of champions to implement collaborative practices cover a wide spectrum. By contextualizing them, we enable decision-makers to integrate them into their own environment.
- These results help to broaden the potential barriers and facilitators to implementing projects using champions. By cross-referencing these factors with the EPIS conceptual framework, we suggest new recommendations for its use.

## Introduction

The increasing complexity of healthcare technologies and the specialized expertise of healthcare professionals underscore the need for precise and effective collaboration [1]. Models for collaborative practice generally agree that healthcare professionals cannot handle a single healthcare episode alone [2, 3]. Effective interprofessional teamwork in healthcare does not require continuous collaboration among all team members, but rather a shared foundation of knowledge, skills and attitudes that facilitate collaborative practice [4]. Moreover, such practices require clear definitions of roles and responsibilities, ongoing communication and the means to monitor patients' progression in their care journey.

Enhancing collaborative practice among health professionals in the workplace can be achieved with continuous training. Such training can be carried out at an individual level (e.g. knowledge and comprehension of roles, understanding group processes), and/or at a group level (e.g. developing a group, working together). The choice of one level over the other depends on the organization and the practices that the institution desire to change, or the evidence-based practices that it wants to implement [5]. From an organizational standpoint, change is achieved through 1) evaluation of current practices, 2) planning, training and implementation, and 3) maintaining and sustaining change [6]. Implementation strategies are numerous and diverse, making a precise taxonomy difficult to establish [7, 8].

Independent of national initiatives aimed at improving collaborative practices, healthcare institutions need to adapt the various conceptual frameworks to their own characteristics, resources and local contexts [6]. They can initiate a process of change at a macro-organizational level (e.g. changes in institutional organization), a meso level (e.g. interdepartmental collaborations) or a micro level (e.g. implementation of communication tools within a service). External experts or internal staff can be involved in the process of improvement as change implementers. External implementers are mandated to intervene in an organization seeking to implement change. Their interventions are generally limited in number, and occur at specific moments in the change process. Internal implementers are asked (or tasked) to implement a given innovation within their own team and are often referred to as “champions”. An integrative review defined the construct of champions as an implementation-related role [9]. People occupying this role 1) are internal to an organization, 2) have intrinsic interest and commitment to implementation, 3) serve as role model for their implementation work, 4) demonstrate enthusiasm, dynamism, persistence, and 5) have strength of conviction [9]. Champions may benefit from training related to innovation or coaching throughout the implementation process [10]. The use of healthcare professionals to champion implementation has the advantage of providing efficient (resources deployed) and effective (prior knowledge of the field) means of setting up collaborative practices [9].

Nevertheless, the use of these local champions implies a great deal of preliminary work to equip them adequately for their role as facilitators or managers of change [11]. Indeed, local champions are seen as critical facilitators of change within the organization, but do not necessarily have the knowledge or skills to manage a process of change in collaborative practices. Similarly, collaborative practices play a substantial role in healthcare routines but are rarely trained beyond the pre-graduate level. There is a need to better understand how champions are involved in the collaborative practice-based innovations, what skills are needed to endorse this role, and which factors influence their implementation process. This article presents the findings of a scoping review on the implementation of collaborative practices involving champions in healthcare settings. The aims of the study were to describe the type of projects involving local champions, their roles, as well as the type of collaborative practices they implemented. We were also interested in identifying the facilitators and barriers local champions faced in implementing collaborative practices.

## Method

Given the exploratory nature and broadness of our research question, we chose to conduct a scoping review. This methodology is particularly suited to overview how champions are defined and their actions are operationalized in multifaceted and context-depended projects. It also enables the inclusion of heterogeneous evidence involving qualitative, quantitative or mixed data, independently of strict inclusion criteria [12]. We used Arksey and O’Malley’s five-step approach to conduct the scoping review [13]. This dynamic and iterative process consists in 1) identifying the research question, 2) identifying relevant articles, 3) selecting the articles, 4) charting the data, and 5) collating, summarizing and reporting the results. We discussed each step in the whole research group and documented the discussions for quality assurance. Our results are presented in accordance with the PRISMA flow diagram and the PRISMA-ScR checklist (Appendix A) [14, 15]. No protocol was published for this research.

### Identifying the research question

Our scoping review focused on the implementation of collaborative practices in healthcare settings using local champions. We explored innovations and champions’ characteristics, collaborative practices, and barriers and facilitators regarding the implementation of collaborative practices with champions.

### Identifying relevant studies

We developed the search strategy (Pubmed and Embase) with the help of an academic librarian. We used three categories of key terms: 1) population (e.g. champion; “early adopter”; innovator), 2) process (e.g. implement*; “continuing education”; adoption), and 3) collaboration (e.g. “collaborat*”; “interprofessional*”; “interdisciplin*”). We tested the relevance and accuracy of the selected key terms by ensuring that key articles published on this topic were included in the search results. We used EndNote (version 21.4) to collect and organize the references. Inclusion criteria were articles published from 01.01.2000 until 01.02.2025, written in English, French or German. Included articles reported collaborative practices (interprofessionality, team development, collaboration, working together), using local champions (early adopters, peer teachers, implementers, healthcare professionals) taking place in healthcare setting (healthcare organizations, healthcare institution, and clinical wards). Using a snowball approach, we added articles from the reference lists if they met the inclusion criteria mentioned above and were not listed in the initial search. Exclusion criteria were initiatives to foster clinical or medical practices, articles about patients or pre-graduated learning, and articles using coaching or mentoring programs. We conducted an initial search in September 2023 and an update in February 2025. The comprehensive search strategy can be found in Appendix B.

### Selecting the studies

We imported all articles in EndNote and deleted duplicates. We conducted a calibration exercise on 20 titles and abstracts to refine the inclusion and exclusion criteria. We discussed and reformulated the criteria using an iterative process. We conducted the articles selection in three phases: 1) based on the titles, 2) based on the abstract, and 3) based on the full text. We imported titles and abstract in a prepared Excel file, enabling article classification as “included”, “exclude”, or “unsure”. Articles labelled as “unsure” were included in the next step of the article selection process. RL randomly attributed the articles in working pairs. Each pair discussed selection screening discrepancies. In case of disagreement, each pair categorized the article as “include”. We applied strict inclusion and exclusion criteria to determine eligibility in each phase. Final extraction between the working pairs reached substantial interrater agreement (0.70 < k ≤ 0.73) [16].

### Charting the data

We first defined the data to be extracted. We included the following information: article publication information (year of publication, author, country), article characteristics (language, aims of the study, study design, approach used, targeted institution, medical specialty, participants), project implementation (framework used, focus on the use of champions, type of champions, role of the champions, training, recruitment, method of implementation, duration, championing program), and facilitators and barriers. RL and NJP independently extracted data and then compared them to ensure the reliability of the adopted approach. Extraction difficulties or discrepancies were resolved by discussion with a third party (MCA) until consensus was reached.

### Collating, summarizing and reporting the results

We mainly obtained qualitative results from the data extraction sheets (Appendix C). We draw from the Lane-Fall and colleagues’ subway analogy to categorize study aims [17]. Included articles fell into the following and nonexclusive categories: a) Pre-Implementation (articles that assess readiness to change, context evaluation or stakeholders analysis in the premises of an implementation project), b) Implementation (articles that describe the implementation processes or features such as use of early adopters, champions), c) Post-Implementation (articles that assess implementation effectiveness or impact), and d) Lessons learned (articles that describe the evidence-based practices issued from a specific implementation strategy or strategies).

For implementation-related topics, we used the taxonomy developed by Andersson, Elg, Idvall and Perseius [18]. It classifies the types of practice-based improvement ideas in health care services into five categories: 1) the organizational process which refers to *the systemic ways in which an organization defines, organizes and implements its activities closely related to process improvement* (i.e. restructuring, change in organizational roles, psychosocial work environment, organizational mapping, patient flow), 2) the evidence and quality which relates *to concepts, methods and principles developed externally* (i.e. support evidence-based practice, quality register work, other quality follow up, national standards and guiding principles), 3) the competence development which refers to the *continuously improvement and development of staff members’ competence* (i.e. auscultation, self-education, practice in realistic environment, training other staff), 4) the process technology that includes *methods and techniques aiming to support different kinds of process-based activities* (i.e. implement new clinical methods and technologies, purchase equipment, supportive IT systems), and finally 5) the proactive patient work which refers to the proactive work *to prevent and minimize hospitalization* (i.e. patient education—self-care, screening risk groups).

We used D’Amour and colleagues’ model and typology of collaboration between professionals in healthcare organizations to categorize implemented collaborative practices [19]. The four interrelated dimensions are 1) shared goals and vision (existence of common goals and their appropriation by the team; goals, client-centered orientation vs other allegiances), 2) internalization (awareness by professionals of their interdependencies and the importance of managing them; trust, mutual acquaintanceship), 3) governance (leadership functions that support collaboration; centrality, leadership, support for innovation, connectivity), and 4) formalization (structuring clinical care; formalization tools, information exchange).

Facilitators and barriers related to implementing collaborative practices in healthcare settings through local champions were categorized using the EPIS framework [20]. This framework is composed of four dimensions, namely outer and inner contexts, and bridging and innovation factors. The outer context describes the environment external to the organization (e.g. governmental policies, patients’ characteristics, and inter-organizational relationships). The inner context refers to the characteristics within an organization (e.g. leadership, organizational structure, internal policies, staffing, and adopters). Bridging factors influence the implementation process and reciprocally (e.g. consumers, academic policies, industry lobbyists). Innovation factors include characteristics of the innovation or EBP characteristics (e.g. fit of the innovation, championing process, communication).

## Results

**Fig. 1:**
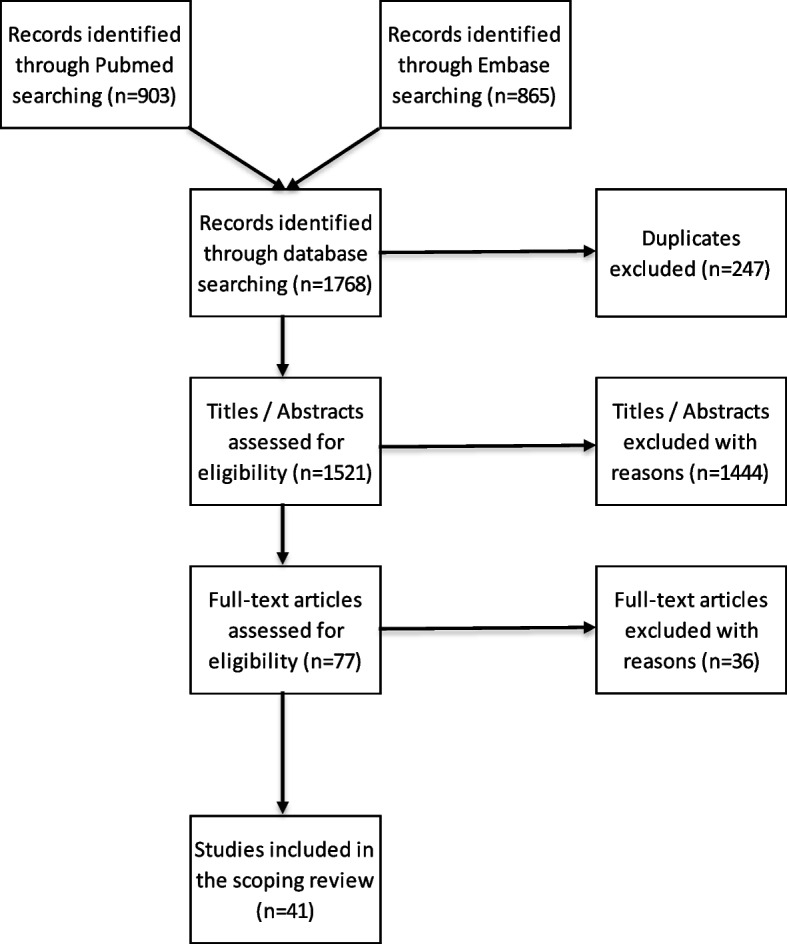
PRISMA flow diagram

The initial search identified 1768 titles of which 247 were duplicates. After screening, we assessed 77 full text articles. We identified 41 articles that met inclusion criteria and underwent full data extraction (Fig. 1).

### Study characteristics

Included articles were conducted in nine countries, mainly USA (*n* = 24; 58%), Canada (*n* = 6; 15%), France (*n* = 3; 7%), and the Netherlands (*n* = 3; 7%). Included articles were published during the last ten years (2014–2024, *n* = 30; 73%). All publications were written in English.

### Study aims

Several articles had single aims: lessons learned (*n* = 14; 34%; [21–34]), post-implementation (*n* = 5; 12%; [35–39]), pre-implementation (*n* = 3; 7%; [40–42]), and implementation (*n* = 3; 7%; [43–45]). Articles with multiple aims included implementation and post-implementation (*n* = 7; 17%; [46–52]), post-implementation and lessons learned (*n* = 4; 10%; [53–56]), implementation and lessons learned (*n* = 3; 7%; [57–59]), and pre-implementation and implementation (*n* = 2; 5%; [60, 61]).

### Study designs

Most of the articles used a qualitative approach (*n* = 24; 59%). Fifteen of them used interviews for data collection [21, 23–26, 32, 35, 41, 42, 44, 53, 55, 58–60]. Two of them used critical review [31, 34] and the last seven used several qualitative methods (e.g. observation, interviews, and documentation analyses) [22, 27, 29, 33, 45, 57, 61]. The mixed method articles (*n* = 14; 34%) used different methods such as interviews (*n* = 7; [28, 30, 39, 40, 49–51]), analyze of clinical data (*n* = 7; [28, 36, 48, 50–52, 56]), surveys (*n* = 5; [30, 36, 37, 40, 49]), observations (*n* = 3; [43, 47, 56]), and critical review (*n* = 3; [37, 39, 40]). There were only three quantitative articles (7%), which exclusively relied on surveys as data collection method [38, 46, 54].

### Study populations

Most articles were conducted in single-type institutions (*n* = 33; 80%) such as hospitals (*n* = 21; [22, 23, 26, 30–32, 36, 41, 43, 45–50, 52, 54, 55, 57, 60, 61]), medical centers (*n* = 10; [27, 28, 33, 35, 37, 39, 40, 42, 53, 58]), or community centers (*n* = 2; [29, 44]). Eight articles (20%) were multi-types such as hospital and community centers (*n* = 1; [51]), hospital and medical center (*n* = 5; [21, 25, 34, 38, 56]), and all three category types (*n* = 2; [24, 59]). Regarding types of medical specialties, articles were mainly conducted in primary care (*n* = 8; 19%; [24, 27–29, 33, 39, 44, 53]), acute care (*n* = 4; 10%; [26, 31, 36, 60]), geriatrics (*n* = 4; 10%; [35, 40, 51, 59]), surgery (*n* = 4; 10%; [34, 46–48]), and others (10%), such as pediatrics [22], psychiatry [43], pharmacy [58] and palliative care [25]. Four articles (10%) were conducted in multi-specialty settings [37, 50, 56, 61] and thirteen articles (31%) did not mention any specialty [21, 23, 30, 32, 38, 41, 42, 45, 49, 52, 54, 55, 57]. Population of interest was mainly interprofessional (*n* = 19; 46%; [22, 24, 26, 27, 29, 32, 33, 35, 38, 39, 41, 42, 45, 50, 52, 55, 56, 60, 61]) or did not specify the population’s professions (*n* = 14; 34%; [21, 23, 28, 31, 36, 40, 44, 46, 47, 49, 51, 54, 57, 59]). Six articles (14%) focused only on nurses [25, 30, 34, 43, 48, 53], one on patients [37] and one on pharmacists [58].

### Champions

The champions’ professions were specified in 30 articles (73%). Ten articles used mono-professional champions such as nurses (*n* = 6; [25, 34, 40, 43, 48, 53]), physicians (*n* = 2; [38, 47]), pharmacists (*n* = 1; [58]) and researchers (*n* = 1; [44]). Seven articles included champions from two professions such as physicians and nurses (*n* = 4; [26, 27, 30, 59]), physicians and administrative staff (*n* = 2; [41, 46]), and social workers and researchers (*n* = 1 [29]). Thirteen articles used championing teams with more than two professions [22, 24, 32, 33, 35, 37, 39, 45, 50, 52, 56, 60, 61]. Eleven articles (27%) did not specify champions’ professions [21, 23, 28, 31, 36, 42, 49, 51, 54, 55, 57].

Champions’ recruitment was mainly done through the identification of potential champions by managers or project leaders (*n* = 13; 32%; [21, 26, 27, 29, 31, 32, 35, 37, 40, 42, 45, 48, 59]). Other recruitment processes were volunteering (*n* = 8; 19%; [30, 36, 38, 39, 43, 52, 60, 61]) and selection/designation of the champion by proximity leaders (*n* = 7; 17%; [24, 33, 41, 44, 46, 51, 58]). Some articles (13%) indicated the use of multiple recruitment modalities such as identification and selection (*n* = 2; [23, 53]), selection and volunteering (*n* = 2; [29, 34]), and identification, selection and volunteering (*n* = 1; [49]). Eight articles (19%) did not specify the recruitment process [22, 25, 47, 50, 54–57].

Champions’ roles were described as manager (*n* = 7; 17%; [21, 32, 34, 42, 46, 47, 51]), staff member (*n* = 4; 10%; [25, 43, 53, 61]), or facilitator (*n* = 2; 5%; [33, 44]). Champions’ roles were also mixed, such as between staff member and manager (*n* = 7; 17%; [22, 26, 27, 35, 40, 48, 49]), and between the three categories (*n* = 13; 32%; [24, 29, 30, 39, 41, 45, 50, 52, 55, 56, 58–60]). Eight articles (19%) did not specify the champions’ role [23, 28, 31, 36–38, 54, 57]. Regarding implementation leadership, champions commonly acted as early adopters or initiators who advocated for new models of care, tools or guidelines (*n* = 7; 17% [21, 22, 29, 37, 40, 42, 59]), facilitators or mentors who mentored the staff and built buy-in (*n* = 6; 15% [26, 34, 43, 48, 49, 52]), and operational coordinators who ensured implementation’s alignment with institutional goals and handled operational challenges (*n* = 7; 17%; [23, 25, 32, 33, 56, 58, 60]). Half of included articles did not specify implementation leadership (*n* = 21; 51%; [24, 27, 28, 30, 31, 35, 36, 38, 39, 41, 44–47, 50, 51, 53–55, 57, 61]). When mentioned, leadership style used by the champions was transformational (i.e. inspiring, setting vision, systemic change) (*n* = 7; [29, 32, 35, 51, 54, 56, 59]), collaborative (i.e. co-design, commitment, teamwork) (*n* = 10; [21, 26, 30, 36, 43, 44, 49, 52, 53, 61]), supportive (i.e. coaching, mentoring) (*n* = 12; [22, 23, 25, 33, 34, 39, 40, 42, 48, 55, 58, 60]), directive (i.e. top-down, regulatory) (*n* = 3; [31, 47, 50]), adaptative (depending on organizational context for multi-site implementation) (*n* = 3; [28, 45, 46]), or mixed (*n* = 4; [24, 27, 37, 38]). There was a strong link between existing clinical or organizational roles and the champions’ recruitment. Champions with clinical expertise such as nurses or physicians led projects directly linked to their (clinical) domain of practice (*n* = 13; [30, 34, 39, 40, 43, 48, 49, 52, 53, 55, 58, 60, 61]). Organizational champions were usually higher-level managers supporting the implementation through, for example, policy alignment, organizational restructuration, or cultural change (*n* = 10; [29, 31–33, 35, 47, 50, 51, 54, 56]). Some articles indicated using both approaches within a championing group (*n* = 7; [21, 22, 24–26, 37, 59]). Of the included articles, 8 (19%) were unclear or did not indicate such links [27, 28, 38, 41, 44–46, 57].

Champions’ training for the implementation’s project was specified in 23 articles (56%). This included formal training (*n* = 11; [25, 27, 33–37, 49, 50, 56, 59]), mentoring (*n* = 7; [22, 42, 44, 46, 47, 55, 58]), quality improvement programs (*n* = 3; [29, 41, 60]), and train-the-trainer models (*n* = 2; [51, 52]).

The way champions were involved in the implementation of collaborative practices (i.e. championing process) was specified in 16 articles (39%). Those articles used on the field training (*n* = 7; [28, 37, 45, 50, 52, 59, 61]), counselling through champions (*n* = 4; [33, 36, 49, 51]), gradual onboarding of people and implemented tools (*n* = 3; [26, 27, 48]), and role modelling of local champions (*n* = 2; [42, 43]). Overall, among included articles, 19 (47.5%) had a clear focus on the impact of champions in the implementation projects [22, 23, 25–30, 33, 34, 41, 42, 47, 49, 50, 53, 58, 60, 61].

### Implementation projects and collaborative practices

Twelve articles (29%) did not specify the duration of the implementation project [21, 23–26, 34, 35, 38, 40, 44, 51, 58]. Four projects (10%) took place over less than a year [46, 52, 57, 61], 10 (24%) between one and two years [22, 30, 39, 41, 45, 48, 49, 53–55], 9 (22%) between two and three years [27, 28, 32, 33, 36, 37, 43, 47, 50], and 6 (15%) more than three years [29, 31, 42, 56, 59, 60] with a maximum at 3 years and half years [31].

Most articles did not mention any use of a theoretical framework for the implementation process. When mentioned (*n* = 13; 32%), the frameworks were: the Consolidated Framework for Implementation Research – CFIR (*n* = 6; [22, 24, 33, 35, 55, 58]), the Plan-Do-Study-Act cycle – PDSA (*n* = 4; [31, 37, 38, 43]), the Explore-Prepare-Implement-Sustain framework – EPIS (*n* = 1; [60]), the Iowa model of evidence-based practices (*n* = 1;), and the mentored implementation model (*n* = 1; [51]).

The use of champions to implement collaborative practices was integrated in different implementation projects as highlighted in Table 1. Topics of implemented projects were divided into the following categories: organizational process (*n* = 11; 27%; [25–27, 29, 38, 40–43, 49, 53]), evidence and quality (*n* = 21; 51%; [22–24, 28, 32, 34–37, 39, 45, 47, 48, 50–52, 54–56, 59, 60]), competence development (*n* = 2; 5%; [30, 46]), process technology (*n* = 6; 15%; [21, 31, 33, 57, 58, 61]), and proactive patient work (*n* = 1; 2%; [44]). Included articles focused primarily on organizational processes, evidence and quality, and process technology. Regarding implementation, topics linked with champions’ professions, mono-professional champions’ projects covered a broader spectrum of topics. The greater the diversity of professions involved in championing teams, the more specific the implementation’s topics were, such as competence development or the implementation of equipment.

**Table 1:** Topics of implementation according to champions’ team professional composition

Champions’ professions	Topic of implementation	Examples
**Mono-professional**	Organizational process	Workflow process or program [38, 43, 53] Staff members' characteristics and roles [25, 40]
	Proactive patient work	Protocols including patients [44]
	Evidence and quality	Support of evidence-based practices using champions [34, 47] Framework to guide health care [48]
	Process technology	Experience with clinical method [58]
**Bi-professional**	Competence development	Continuous training curriculum [46] Knowledge translation project [30]
	Organizational process	Staff members' characteristics and roles [26, 27, 29, 41]
	Evidence and quality	Framework to guide health care [59]
**Multi-professional**	Evidence and quality	Framework to guide health care [32, 35, 37, 50] Support of evidence-based practices using champions [22, 24, 39, 52] Quality monitoring program and processes [45, 56, 60]
	Process technology	Dashboard and equipment [33, 61]
**NA**	Organizational process	Work environment and climate [49] Staff members' characteristics and roles [42]
	Evidence and quality	Quality monitoring program and processes [54] Support of evidence-based practices using [23] Framework to guide health care [28, 36, 51, 55]
	Process technology	Support for innovators [21] Dashboard and equipment [31, 57]

Table 2 shows the different types of collaborative practices implemented with the help of champions within their respective topics of implementation. Since most included articles reported the implementation of a bundle of collaborative practices, they fell into one or more of the following categories: shared goals and vision (*n* = 11), internalization (*n* = 18), governance (*n* = 22), and formalization (*n* = 21). Thirteen articles (32%) had a focus on one of these categories [22, 25, 27–29, 31, 37, 43, 45, 46, 50, 52, 61], 23 (56%) on two categories [26, 30, 32–36, 39–42, 44, 48, 49, 51, 53–60], and 5 (12%) on three categories [21, 23, 24, 38, 47]. The number of categories covered in each article was not related to the span of the innovation (i.e. national, regional or local), the project duration, the study’s characteristics or the implementation topics. There was no predominant collaborative practice among included articles.

**Table 2:** Types of implemented collaborative practices according to topics of implementation

Topic of implementation	Collaborative practices	Examples
**Organizational process**	Shared goals and vision	Building inter-/intra-organizational partnership, community embeddedness [42] Understanding of organizational goals and internal culture [42]
	Internalization	Multi-professional meetings and collaboration across discipline [26, 29, 49, 53] Interdisciplinary rounds and practices [38, 53] Relationship development and inter-/intra-organizational connections [26, 29, 41] Team practices in multi-professional teams [38, 41]
	Governance	Professional development such as clinical and continuing education [40, 41, 49] Guidance, consulting, feedback and monitoring [25, 38, 40] Change management activities – creating teams, managing meetings, coordinating work [27, 38, 49] Group webinars, community of practice, local community [25, 41, 42, 44, 47]
	Formalization	Pair/peer work models and multi-professional teams [26, 43, 53] Roles and responsibilities, tasks’ standardization [40, 43] Protocols’ optimization, working models, activity redesigning and collaborative routines, customization [26, 38]
**Evidence and quality**	Shared goals and vision	Building inter-/intra-organizational partnership, community embeddedness [23, 39, 52, 54] Understanding of organizational goals and internal culture [23] Mutual alignment, shared team understanding [34, 39, 52]
	Internalization	Multi-professional meetings and collaboration across discipline [23, 24, 47, 48, 54] Team practices in multi-professional teams [28, 32, 54] Interdisciplinary rounds and practices [32, 48] Relationship development and inter-/intra-organizational connections [23, 24, 28, 39, 56]
	Governance	Leadership style and support for the implementation [22, 35, 37, 55] Guidance, consulting, feedback and monitoring [24, 37, 47, 50, 60] Group webinars, community of practice, local community [24, 34, 47, 51, 55] Change management activities – creating teams, managing meetings, coordinating work [36, 45, 50, 55] Professional development such as clinical and continuing education [24, 37, 45, 59]
	Formalization	Pair/peer work models and multi-professional teams [23, 32, 35, 47, 48, 60] Protocols’ optimization, working models, activity redesigning and collaborative routines, customization [23, 24, 32, 36, 51, 59, 60] Roles and responsibilities, tasks’ standardization [56, 60] Program/hospital communication strategy [24, 55, 56, 59, 60]
**Process technology**	Shared goals and vision	Use of evidence [21] Building inter-/intra-organizational partnership, community embeddedness [21] Understanding of organizational goals and internal culture [57, 58] Mutual alignment, shared team understanding [57, 58]
	Internalization	Relationship development and inter-/intra-organizational connections [21, 58] Team practices in multi-professional teams [31, 33, 58] Interdisciplinary rounds and practices [31] Multi-professional meetings and collaboration across discipline [33]
	Governance	Group webinars, community of practice, local community [61] Change management activities – creating teams, managing meetings, coordinating work [61]
	Formalization	Pair/peer work models and multi-professional teams [21, 33] Roles and responsibilities, tasks’ standardization [57] Protocols’ optimization, working models, activity redesigning and collaborative routines, customization [33, 57]
**Competence development**	Shared goals and vision	Building inter-/intra-organizational partnership, community embeddedness [46] Mutual alignment, shared team understanding [46]
	Internalization	Multi-professional meetings and collaboration across discipline [30] Team practices in multi-professional teams [30]
	Formalization	Roles and responsibilities, tasks’ standardization [30] Program/hospital communication strategy [30]
**Proactive patient work**	Shared goals and vision	Mutual alignment, shared team understanding [44]
	Governance	Group webinars, community of practice, local community [44]

Of the included articles, 16 (39%) reported outcomes and impact from the implementation process of studies including post-implementation evaluation. From those articles, many showed strong positive change after the innovation’s implementation using champions such as overall increase in evaluation’s scores [46, 50, 51], reduction of infections or length of stay [48], or improvement in standards [36, 49], professional [52] or project management [37, 38] competency. Champions also facilitated culture change by integrating new norms in the activities [55] or positive externalities such as improved interprofessional relations [50]. Few articles indicated moderate or partial success in the implementation such as partial innovation’s adoption by the staff [39, 53], fragmented implementation, or modest improvement in evaluation’s scores [36, 54, 56].

### Facilitators and barriers

Barriers and facilitators with their related articles are reported in Appendix D. Since some authors also mentioned potential factors influencing the implementation of collaborative practices without highlighting their positive or negative impact within their research, we added a neutral category. Details of the items composing the barriers and facilitators identified for each construct are summarized in the following sections.

The barriers and facilitators linked with the internal context were the most numerous. Internal context refers to the characteristics within the organization such as leadership, organizational structure and policies, and staffing. Most articles emphasized the fact that champions and local leaders needed to be fully committed for the innovation. The personal qualities of champions, such as formal authority, role modelling, influence, inspiration, persuasiveness and motivation were highlighted. The characteristics of local working environments included a supportive interprofessional culture, availability of resources as well as support for change. The size of the institutional or working organization was both seen as a facilitator or a barrier. Project monitoring was considered as a critical component, which included refined planning, project staging, recognizing and awarding change effort, project reporting and sharing artefacts that make change sustainable, tangible and visible. Many articles identified staff turnover as a major barrier to the implementation of innovation. Project-oriented trainings and ongoing training processes were perceived as essential to equip both champions and staff members with the requested skills to implement the innovation effectively. This allowed involved professionals to co-develop a shared vision and mutual understanding. Finally, individual characteristics, primarily those of staff members and sometimes of champions, included passion, motivation, commitment and openness to collaborative practices. Personal barriers were reported when individuals did not identify with the project, felt their professional identity was threatened or became frustrated with the implementation process or subject.

The innovation factors highlight both the characteristics of the innovation itself and its alignment with the context. Several of the included articles focused more on the innovation characteristics and how they matched local needs than on the profiles and behaviors of its developers. Since most implementation projects were embedded with already existing evidence-based recommendations, interactions with developers were not always possible. However, many articles considered it important to integrate staff representatives in the innovation development team to ensure mutual understanding of the needs in a multi-professional manner, whenever possible. There was a common agreement among most articles that the innovation should provide adaptable tools and processes, be flexible and easy to understand, and allow local champions to monitor and report about changes using quantitative measures and evidence.

Regarding outer context, there were several barriers and facilitators. The outer context describes the environment external to the organization such as policies, leadership, patients and other organizations. Articulation with national or regional projects and current local practices was perceived as helpful. Major crises were reported to have both positive and negative impacts, depending on where resources were allocated. Similarly, financial support was considered as a facilitator or a barrier depending on its presence. Direct implication or indirect commitment of decision-makers in the field was a strong facilitator. The presence of inter-organizational environment and networks was often mentioned as a facilitator since partnerships with external organizations and representatives helped coordinate tasks and pool implementation efforts. Project that lacked clear links with regional or national objectives, or with evidence-based practices, as well as those with implementation processes extending beyond the scope of the institution faced challenges in communicating with partners.

The final level in the EPIS framework is referred to as bridging factors. Elements influencing the implementation process—and are influenced by it in return—such as consumers, academics and lobbyists, are considered as bridging factors. Most articles highlighted bridging factors as facilitators. A scientific approach and reliance on evidence-based practices were considered essential for seamless implementation. Building partnerships with scholars helped champions assess their own practices and develop effective implementation processes. Regular communication between champions and innovation experts was described as necessary to build trust. Co-designing an adaptable and needs-centered innovation also strengthened the collaboration between champions and experts. As investigators, we experienced difficulties in discriminating between intermediate purveyors, innovation leaders and local leaders. Nevertheless, several articles highlighted the importance of an intermediary link between the innovation and the end-users to manage change, respectively to promote, support and sustain it over time. Intermediaries played a central role as they had knowledge of the project; they were therefore the most likely to be able to support local agents of change, and prevent the latter from being overwhelmed by the innovation or its implementation process.

## Discussion

Our scoping review aimed at highlighting current practices in the use of champions to implement collaborative practices within the healthcare setting. We explored the characteristics of both innovations and champions, the type of collaborative practices implemented, as well as the related barriers and facilitators. Our results showed that included articles were mainly monocentric, qualitative, and focused on the process of implementation. A third of the articles explicitly used a theoretical framework for the implementation process. The implemented innovations were mostly linked to organizational processes, development, and evidence-based and quality improvement. More than half of the studies aimed at implementing at least two types of collaborative practices. Less than half of the articles had a clear focus on the impact of champions in the implementation. Champions were mainly appointed by the institution rather than volunteering. They came from a variety of specialties and worked in interprofessional teams. Authors provided little information about their specific roles and positions within the institution and the training provided to work as a champion. Finally, the included articles revealed a rich variety of factors influencing the success or failure of implementation projects. From an individual to an organizational or national perspectives, most facilitators and barriers regarding the implementation of collaborative practices referred to the inner context.

Most included articles were qualitative and monocentric and only few of them used evidence-based frameworks to document their implementation process. This is surprising since such frameworks are specifically designed to consider contextual specificities and experiences when implementing quality improvements projects and/or collaborative practices [62, 63]. They offer a clear step-by step model that provides structure and direction, and that supports vertical and horizontal collaboration in a given context. They enhance collection and use of data to assess and monitor changes and encourage local adaptation and sustainability [64, 65]. Although best practices in implementation science highlight the importance of project evaluation, only a few articles measured the effectiveness of collaborative practices led by champions. Monitoring the effects of an implementation needs time and precise indicators. A suitable approach resides in using longitudinal and mixed methods in order to display contextualized evidence [66]. Such evidence will support healthcare institutions in making decisions about what innovations to adopt, how to implement them, who should lead the process (champion), and why they matter in their specific context.

Champions were mainly identified and selected within the institution. However, it remained difficult to differentiate their roles and implications in the implementation process in several studies. It was unclear if the champions were institutional leaders responsible for the innovation, appointed champions leading the project, or local champions managing change on the field. This confusion may be explained by the fact that the responsibilities for implementation were often vague, and information about the champions’ roles and background was often lacking. Champions are typically defined by their specific interest in, and knowledge of, the implementation subject [9]. They can be professionals from any hierarchical level. However, project champions do not all have the same power to lead change within the organization. To be successful, champions must be able to influence peers, build trust, communicate across professional groups, and adapt to institutional barriers while fostering collaboration [67]. It requires a variety of broad interpersonal, organizational, and problem-solving skills that need to be specified, formalized and trained. Their leadership is often linked with institutional structure and embedded in the development of their professional identity [68]. Since most champions were clinicians or operational (administrators, researchers), their formal roles conferred them credibility and authority. Their expertise-based leadership enabled them to influence peers and mobilize organizational resources effectively [69]. Moreover, the dominant leadership styles observed were participatory and facilitative, consistent with transformational leadership theory, which emphasizes proactive, supportive, and perseverant leadership behaviors [68, 70]. Successful implementation depends not only on the presence of champions but on their ability to lead through relational influence, contextual awareness, and dynamic systems [71]. Again, the use of a framework may help promotors of champion-led projects to better understand how the different levels of management (e.g. the dimensions of the EPIS framework) interact with each other. This can help clarify at which level champions should intervene and what forms of support are expected from other managers. The absence of information about the champions in the implementation process leads to poor generalizability and replicability of the results [72–74] and highlight the need to standardize the need of reporting of champions' role in implementing evidence-based practices [10, 75, 76].

Another key theme about champions is their training. While half of the integrated articles referenced champions’ trainings, descriptions were often vague and lacked detail. This is of concern since adequate training remains critical for developing implementation competence and fostering collaboration among champions. Given that local champions often come from diverse professional backgrounds, establishing a shared understanding and a common ground between champions is critical [77]. Joint training offers several advantages: it facilitates the acquisition of project-specific knowledge and cross-functional management skills needed to act as change agents; it promotes the development of a shared mental model among champions; and it strengthens interpersonal relationships and teamwork within the group. Bringing champions together for collective reflection not only enhances mutual understanding, but also builds team cohesion and clarifies roles around common goals within the implementation strategy [78–80]. However, implementing such transversal training at the institutional level requires a comprehensive strategy. This strategy should include the allocation of protected training time, supervision, and field-based support to ensure successful implementation and effective skill transfer [79]. To optimize this approach, institutions must also clearly communicate their objectives, outline the methods used, and specify the support expected from both staff and management [77].

Most recommendations regarding the implementation of collaborative practices were linked to the organizations’ inner context. This is not surprising given the focus on internal championing. Included articles underscored the importance of local leadership in facilitating the adoption of innovation. The commitment of leaders is a critical determinant of successful innovation [81]. Leaders can inspire and motivate staff, as well as create a shared vision, while fostering a sense of ownership and responsibility for the innovation process. Also, leaders may influence the working environment within the organization. A culture that supports interprofessional collaboration, coupled with adequate resources and organizational support for change, is known to be essential for promoting innovation [23, 82]. Interestingly, the size of the organization was identified as both a facilitator and a barrier. While larger organizations may have more resources, they can be hindered by bureaucracy and complexity. The size and structure of an organization influence the flexibility and adaptability required for successful innovation [69]. Policies, financial support, and regional or national projects were seen as both facilitators and barriers, depending on their alignment with the innovation's goals. The commitment of external stakeholders, such as policymakers and decision-makers, in the implementation process can serve as an enabler of innovation by securing the political and financial support needed to sustain change efforts [83]. Additionally, the presence of inter-organizational networks is known to facilitate collaboration and pooling of resources, which can enhance the collective capacity to implement innovations [84]. These elements show that a clear alignment between local projects and broader national or regional priorities is needed to ensure communication and coordination. Innovations, especially when they involving multiple organizations, must be integrated into wider systems and policies to be successful [85]. This aligns with the notion of policy coherence in implementation science, where integration of local innovations within a broader policy framework is key to success.

### Limitations

There are several limitations to this scoping review. The first limitation of this study was the difficulty in discriminating between roles such as intermediaries, local leaders, and innovation experts. Future research should explore and clearly define the specific roles these individuals play in the innovation process and how their interactions influence the implementation outcomes. Secondly, we used the EPIS framework to identify barriers and facilitators. It is possible that some barriers or facilitators were not taken into account. Nevertheless, given the proximity of conceptual frameworks such as the Consolidated Framework for Implementation Research (CFIR), Plan-Do-Study-Act (PDSA) cycles or Identify, Measure, Analyze, Design, Implement, Measure (IMADIM) process to guide the implementation of innovations, we believe the framework provided a sufficiently exhaustive view of what factors support or hinder the implementation of collaborative practices in the care setting. Finally, we focused on the implementation of collaborative practices in healthcare and therefore did not compare practices carried out in other areas of activity.

### Recommendations

Based on the results of this scoping review, we provide a list of recommendations for both practice and research when involving champions in the implementation of collaborative practice projects (Table 3). Conceptual implementation frameworks highlight the process and the steps for a refined planning. For example, the EPIS framework suggests four steps for the implementation, namely Preparation (evaluate needs and potential innovation fit), Exploration (planning/outreach regarding innovation), Implementation (leadership and support for innovation), and Sustainment (innovation quality assurance) [20]. Leaders’ commitment and involvement in the implementation process is important to advocate for and support change. Collecting both quantitative and qualitative data throughout the process to define and structure the project follow-up with feedback mechanisms, is key. As the idea is to maintain changes over time, ongoing training plays an important role for both existing healthcare professionals and those joining the organization in the future. Some of these suggestions are in line with recommendations of good practices in the implementation of healthcare projects [86–88], but others are more specific to championing itself. An extensive list of recommendations made by each study included in the review and organized through the four dimensions of the EPIS framework is available in Appendix E.

**Table 3:** Recommendations for research and practice regarding the implementation of collaborative practices in healthcare settings using champions

For Research	For Practice
• Defining the profile, background, roles and scope of the champions • Defining the competence and skills required for champions to be successful in the implementation and sustainment of projects • Conducting longitudinal studies to measure impact of champions over time • Collecting mixed outcomes • Referring to an implementation conceptual framework and highlighting how the different dimensions interact with each other’s • Creating consolidated championing guides for the implementation using reported implementations to ensure effective outcome evaluation	• Identifying and choosing champions who are well established and recognized in the field • Building interprofessional championing teams to enhance collaborative practices • Using a conceptual framework to guide the implementation process through different phases such as: ◦ Exploration of the needs; ◦ Preparation for the implementation and training; ◦ Support during the implementation; ◦ Outcome measurement to ensure quality and change sustainment • Stimulating and reinforcing the implementation project with exchanges and collaboration with externs and other institutions • Having designated project managers or coordinators with leadership skills to monitor progress and support champions

## Conclusion

This study highlights the multifaceted roles of champions in the implementation of innovation in a healthcare setting. Although champions are recognized for their ability to drive change, there is limited consensus about how they are selected, trained, and positioned within their institution. Implementing collaborative practices with championing support remains mainly self-made without the support of implementation guidelines. Organizations seeking to implement innovations should use champions in multi-professional teams, prioritize leadership commitment, cultivate supportive environments, invest in training and development, and engage with external networks. Furthermore, careful attention to the adaptability and local fit of innovations, coupled with strong managerial support, is crucial for ensuring the sustainability and effectiveness of healthcare innovations. As healthcare systems continue to innovate, future research must address these gaps by exploring the impact of champions on implementation more systematically, clarifying their roles, and integrating theoretical frameworks to enhance the effectiveness and sustainability of collaborative practice innovations.

## Supplementary Information

Supplementary Material 1. Appendix A PRISMA-ScR checklist

Supplementary Material 2. Appendix B Search terms and search strategy (PubMed + Embase)

Supplementary Material 3. Appendix C Data Extraction

Supplementary Material 4. Appendix D Summary of barriers and facilitators highlighted with EPIS framework

Supplementary Material 5. Appendix E Summary of recommendations highlighted with EPIS framework

## Data Availability

The datasets generated and/or analyzed during the current study are available in the appendix.
