# Supplementary material for: Implementing collaborative practices in healthcare settings using champions: a scoping review
Source: Implement Sci. 2025 Nov 4;20:48. doi: 10.1186/s13012-025-01463-2 (PMC12584293; doi:10.1186/s13012-025-01463-2)
Supplement: Supplementary file 2 — Supplementary Material 2. Appendix B Search terms and search strategy (PubMed + Embase) [file 13012_2025_1463_MOESM2_ESM.docx]

**Appendix B: Search terms and search strategy (PubMed + Embase)**

**Summary of research terms**

| Concept | Population | Process | Collaboration |
| --- | --- | --- | --- |
| Free terms   - Synonyms - Near terms | champion  “Early adopter”  “First mover”  “Master trainer”  innovator | “Continuing education”  “framework”  Implement*  Adaptation  Adoption  “adoption of practices”  “evidence-based practices”  “on the job training”  “Peer-teach” | Collaborat*  Interprofessional*  Multiprofessional*  Interdisciplin*  Multidisciplin*  “collaborative care team”  Team* |
| MeSH  (PubMed) | Peer group  Community-based participatory research | Implementation science  Change management  Quality improvement  Delivery of health care  In service training | Interprofessional relations  Crew resource management, healthcare  Interprofessional education  Cooperative behavior |
| Emtree  (EMBASE) | Innovator  Peer group | Implementation science  Change management  Quality improvement  In service training  Peer teaching  Health care quality | Multidisciplinary team  Interdisciplinary education  Interprofessional education  cooperation |

**PubMed search strategy**

(peer group[MeSH Terms] OR community based participatory research[MeSH Terms] OR champion[Title/Abstract] OR "early adopter*"[Title/Abstract] OR "first mover"[Title/Abstract] OR "master trainer*"[Title/Abstract] OR "innovator*"[Title/Abstract]) AND (implementation science[MeSH Terms] OR change management[MeSH Terms] OR quality improvement[MeSH Terms] OR delivery of health care[MeSH Terms] OR inservice training[MeSH Terms] OR "continuing education"[Title/Abstract] OR teamstepps[Title/Abstract] OR "EPIS framework"[Title/Abstract] OR implement*[Title/Abstract] OR adaptation[Title/Abstract] OR "adoption of practice"[Title/Abstract] OR "adoption of evidence-based practice"[Title/Abstract] OR "peer teach"[Title/Abstract] OR "change management"[Title/Abstract] OR "quality improvement"[Title/Abstract] OR "delivery of health care"[Title/Abstract] OR "inservice training"[Title/Abstract]) AND (interprofessional relations[MeSH Terms] OR crew resource management health care[MeSH Terms] OR interprofessional education[MeSH Terms] OR cooperative behavior[MeSH Terms] OR collaborat*[Title/Abstract] OR interprofessional*[Title/Abstract] OR multiprofessional*[Title/Abstract] OR interdisciplin*[Title/Abstract] OR multidisciplin*[Title/Abstract] OR "collaborative care team"[Title/Abstract] OR "crew resource management"[Title/Abstract] OR "cooperative behavior"[Title/Abstract])

Filters: from 2000 – 2025

**Embase search strategy**

('innovator'/exp OR ‘peer group'/exp OR 'champion':ab,kw,ti OR 'early adopter':ab,kw,ti OR 'first mover':ab,kw,ti OR 'master trainer':ab,kw,ti OR 'peer group':ab,kw,ti OR 'innovator':ab,kw,ti) AND ('implementation science'/exp OR 'change management'/exp OR 'quality improvement'/exp OR 'in service training'/exp OR 'peer teaching'/exp OR 'health care quality'/exp OR 'continuing education':ab,kw,ti OR 'teamstepps':ab,kw,ti OR 'epis framework':ab,kw,ti OR 'implement':ab,kw,ti OR 'adaptation':ab,kw,ti OR (adoption* NEAR/3 practice):ab,kw,ti OR 'on the job training':ab,kw,ti OR 'peer teach':ab,kw,ti OR 'change management':ab,kw,ti OR 'quality improvement':ab,kw,ti OR 'delivery of healthcare':ab,kw,ti OR 'in service training':ab,kw,ti) AND ('multidisciplinary team'/exp OR 'interdisciplinary education'/exp OR 'interprofessional education'/exp OR 'cooperation'/exp OR 'collaborat*':ab,kw,ti OR 'interprofessional*':ab,kw,ti OR 'multiprofessional*':ab,kw,ti OR 'interdisciplin*':ab,kw,ti OR 'multidisciplin*':ab,kw,ti OR 'cooperation':ab,kw,ti) AND [2000-2025]/py

**Exclusion criteria**

1. The study does not concern collaborative practices (working together). E.g. measures to improve clinical practices.
2. The study does not concern healthcare professionals. E.g. student learning, patient learning.
3. The study does not use the “champions” / early adopters / peer teaching model. E.g. coaching or mentoring.

**Inclusion criteria**

1. The study concerns the improvement of collaborative practices, even if this is not the outcome measured.
2. The study concerns healthcare professionals of all professions.
3. The study uses champions / early adopters as a means of change.
