# Supplementary material for: Implementing collaborative practices in healthcare settings using champions: a scoping review
Source: Implement Sci. 2025 Nov 4;20:48. doi: 10.1186/s13012-025-01463-2 (PMC12584293; doi:10.1186/s13012-025-01463-2)
Supplement: Supplementary file 4 — Supplementary Material 4. Appendix D Summary of barriers and facilitators highlighted with EPIS framework [file 13012_2025_1463_MOESM4_ESM.docx]

**Appendix D: Summary of barriers and facilitators highlighted with EPIS framework**

| Dimension | Construct | Barrier | Neutral | Facilitator |
| --- | --- | --- | --- | --- |
| Outer context | Service environment / policies | - Single profession focus of regional / national policies, affiliations’ expectancies ^1,2^ - One-size-fits-all policies ^2,3^ - Lack of national / regional implementation mandate ^3^ - Innovation challenge the core business of national institutions ^4^ - Fragmented landscape of accountability, stakeholders’ activities non alignment ^1,4,5^ - Disincentives from insurance payment policies ^6,7^ - Unfavorable general context (major crisis, competing priorities, poor infrastructures) ^3,8,9^ - Administrative burden ^10^ | - Certification and label of quality ^10,11^ - Designing of policies shaping the implementation ^12-15^ - Identified / designated policy representative ^5^ - Consideration of systemic impact of the innovation ^1^ | - Momentum given by regional / national policies or programs, innovation spread ^2,3,15-23^ - Regulatory bodies to shapes practices, formal guidelines ^1,4,21^ - Innovation reflects dominant ideological beliefs, consistence with current ideas ^4,24^ - Favorable general context (major crisis, necessity of action) ^19,24^ |
|  | Funding / contracting | - Constrained economic climate ^4^ - Difficulties of reimbursement for new services ^3,7^ - Inadequate incentives ^3^ | - Availability of financial resources ^10,14,25^ | - Funding source available (grant) ^3,5,15,18-20,26,27^ - Financial incentives or participation ^3,13,24^ - Dedicated resources for implementation projects and innovations, philanthropic organizations ^1,28,29^ |
|  | Leadership | - Lack of clear definition of responsibilities between stakeholders ^10^ - Lack of integrity, working for oneself, unwillingness to support change ^8,26^ | - Governance is aligned with actual policies and guidelines ^10^ | - Implication of senior / top management and decision makers, guidance, executives’ support ^3,4,10,12,15,22,24,26,27,30,31^ - Commitment of national program’s representatives, participation of health system leadership ^10,13,15,18,19,30^ - Trust in local leaders, given control to local champions, extra-organizational buy-in ^1,8,18,26,27^ |
|  | Inter-organizational environment and networks | - Lack of experience with inter-organizational collaboration, animosity, poor communication ^1,6,8,24,26,32,33^ - Lack of inter-organizational communication (visiting symposium, transfer of information, inter organizational training’s coordination, inter-organizational comparison) ^3,6,20,32^ - High confidentiality hindering communication ^5^ - Lack of interest in the innovation of extra-organizational partners, low support ^3,24,33^ | - Promotion of proactive communication between practitioners, linkages ^6,18,33^ - Subcontractors organization, clear inter-organizational responsibilities, support ^10,14,17^ - Early integration of healthcare partners in the implementation process ^10,20^ - Inspiration from other sites or partners, not reinventing the wheel ^1^ | - Inter-organizational innovation’ s dissemination (symposium, congress, summit, rolling-out the innovation, newsletters) ^4,6,15,18-20,22,27^ - Existing or emergent strong inter-organizational connections ^1,3-5,10-12,16-19,22,24,27^ - Partners’ engagement and support for the implementation (data exchange, onsite visits, trust, peer support, data management) ^1,4,6,15-17,19,20,22,24,26,34,35^ |
|  | Patient / client characteristics | - Patient’s reluctance to the innovation ^2,32^ - Patients’ condition inadequate with the innovation ^32^ | - Variety of patients’ needs or characteristics ^6,32,33,35^ | - Detailing needs of patients and families ^36^ - Designated facilitation teams ^32,36^ - Access to healthcare data ^5,19^ - Interest of patients in the innovation ^24^ |
|  | Patient / client advocacy |  | - Resources and toolkits for / from patients initiative ^20^ | - Partners’ interest in novelty, participation in the innovation ^4,18^ - Partners’ innovation promotion, marketing ^4,18,24^ |
| Innovation factors | Innovation / EBP developers | - Lack of involvement of end-users in the innovation development, different goals ^10,36^ - Lack of project ownership from the end-users, strong control from vendor, lack of flexibility / developers (inefficiencies) ^10,15^ | - External innovation team ^5,15^ - Interdisciplinary or interprofessional development team ^15,32,34,35^ | - Understanding of the local needs, vision, experience with the organization (lenght) ^1,15,16,22,36^ - Representative group of end-users, multi-professional, designated coordination / innovation team, cutting through boundaries, autonomy ^5,12,16-18,20,24,27,32,34,36-38^ - Meeting the field, exchanges and formative evaluation, pilot testing ^1,5,20,32,34^ - Preprinted standards, material ^15,20,32^ |
|  | Innovation / EBP characteristics | - Lack of quantitative evidence to support implementation efforts, lack of compliance with current specifications / guidelines ^4,10,12,32^ - Complexity of the innovation, lack of information ^3,5,10,12,34,36^ - Innovation cost, opportunity cost ^31,32^ - Prioritization issues, high “protocolization” ^10,32^ | - Perception of the innovation, increasing awareness ^5,39^ - Innovation delimitations and objectives ^10,17,18,21,28,31,34,35,40^ - Variety in implantation strategies, multifaceted program ^9,17^ - Integrated model for implementation ^14,20^ - Considering survey fatigue ^5^ - Training protocols ^5,20^ - Evidence-based tools, templates ^5,17,18,20,22,28,32,34^ - Dynamic metrics and program coverage ^1,17,32^ | - Balancing capacity of the innovation (adaptability, re-invention, material, bundle) ^2,4,10,12,16,20,24,27,32,35-37^ - Easiness to understand, proximity with current practices ^12,22,27,31,32,34-39^ - Innovation as a model of quality improvement / standards, EBP, systemic approach, protocols and flowcharts ^1,4,12,15,16,20,24,27,28,31,35,39^ - Possibility to collect quantitative evidence (impact reporting) ^4,15-17,20,24,27,28,32,35^ - Improving quality of life of users, usability ^11,20,34^ - Prioritization, planning of the innovation, “game’s rules” ^1,5,12,18,28,36,37^ |
|  | Innovation / EBP fit | - Absence of change sustainment ^2,12,17^ - Innovation is too specific / not include the whole work process ^2,10,12,32^ - Poor patient’s fit or awareness ^2,24^ - Complexity of project monitoring, lack of oversight ^5,8,10,12,17,39^ - Significant difference with current practice or professional background ^4,9,27^ - Lack of on-boarding program ^10^ - Difficulties in accessing material relevant for the innovation ^24,32^ | - Promotion of interprofessional care ^2^ - Implementation staggering ^10^ - Determining innovation and its implementation based on the local needs, collecting local data ^5,8,12,16^ - Community embeddedness ^8^ - Framing function of the project ^16^ | - Innovation meeting the needs of professionals, perceived added value, tailoring innovation for the needs, customization, embeddedness in routines ^2,5,8,10,12,14-20,22,24,27,28,30,32,34-36^ - Community participation, involvement of end-users, bottom-up approach ^1,2,5,6,17,18,22,24,30,34-36,38^ - Highlighting the need for change, mutual understanding ^1,4,5,16,17,27,30,34^ - Formative evaluation, testing ^10,32^ - Proactive communication, explaining expectations ^5,12,17,20,24,34,35,37^ |
| Bridging factors | Community-academic partnerships | - Lack of interactions between end-users and expert team ^6^ - Resistance and complexity in managing expectations ^10,27^ - Concurrent projects implementations ^10^ | - Integrated practices between healthcare practices (primary, specialty), community driven change ^5,8,18,35^ - Implementation strategy, marketing, communication ^1,34^ | - Communication through academic publication ^4^ - Incorporating research findings into clinical practice, monitoring literature, scientific backing ^11,16,18,20,22,32,35^ - Contextual inquiry, exploration pre-implementation, deep understanding of the context, gap analysis ^1,10,15,18,19,22,30,31,34,36^ - Regular communication between research group and end-users, availability, collaboration trust ^1,6,15,20,24,27,34-36,38^ - Participative innovation development and co-design ^1,5,10,15,16,22,27,33,34,36,38^ - Support from corporate healthcare system, perspectives integration (guidelines, objectives) ^1,15,16,27^ |
|  | Purveyors / Intermediaries | - Poor responsibilities definition between experts and end-users ^6^ - Complexity to reach or discuss with purveyors ^10^ - Lack of motivation / commitment of the purveyors ^10^ - Insufficient planning from the purveyors ^1^ | - Naturally designated local champions for the innovation ^18,26^ - Listening to the field, project tailoring ^1^ - Collaboration with academics for the monitoring / evaluation ^18,27^ - Facilitating through purveyors or innovators ^34^ | - Presence of clinical / field champions ^10,11,13,17,22,27,32,35,39^ - Support in the use of the guidelines, external centralized training ^9,11,15,18,22,24,33,37^ - Expertise in research methods / implementation, experience ^12,15,16,18,20,21,33,34,36^ - Implication of researchers or external representatives (collecting data, monitoring, support, co-management) ^1,6,15,16,18-20,27,31,33,34^ - Identification and implication of local champions, merging champions into existing teams ^9,13,15,17,20,22,26,27,32,35,38^ |
| Inner context | Organizational characteristics | - Poor awareness of other professions competencies, roles and skills, poor interpersonal connections ^2,6,10,12,31,38^ - Lack of institutional support (resources, guidance, time, recognition, protection) ^3-5,8,12,17,21,23,26,33,38^ - Organizational characteristics (size (too big, too small), siloing, poor relationships or communication processes, poor learning climate, workflow incompatibility) ^7,12,21,29,32,38^ - Poor support from decision-makers ^7,33^ - Competing priorities within the organization ^3,10,12,22,23,26^ - Lack of common ground for comparison ^20^ - Ceiling effect ^33^ | - Existing support for monitoring ^25^ - Organizational characteristics, assessment (size, type, culture) ^1,8,18,25,29,33^ - Business model ^24^ - Addressing service redesign and workforce issues, guidance ^8,12,29^ | - Workflow knowledge and optimization / maximization of professional contribution ^2,6,7,12,22,24,26,31,33^ - Interprofessional collegiality, trust, respect ^1,2,6,22,27,31,33^ - Availability of monitoring, infrastructure, allocation of resources ^10,13,15,17,20,22,24,32,33,37,39^ - Institutional receptiveness / openness / readiness (culture, preparation of change, support, alignment with the innovation) ^1,4,6-8,18,22-26,28,30,33,37^ - Institutional organization (presence of QI team) ^31,41^ - Implementation strategy including stakeholders / end-users ^1,3,19,24,30^ - Small hospital size ^27^ |
|  | Leadership | - Absence / departure of leaders to champion change, lack of formal authority ^1,2,6-8,12,21,23,32,33,37^ - Different educational background between innovators and leaders ^3,4,6^ - Concerns, skepticism of leaders or opinion leaders, absence of consensus between leaders, lack of consideration of different perspectives, threat to oneself ^7,10,12,26,31-33^ - Lack of ownership or training / knowledge, lack of recognition by end-users, unclear responsibilities, competition ^3,7,9,12,31,33,38^ - Lack of dedicated time / resources of leaders to manage change ^12,23^ - Lack of cooperative spirit, “soloists” ^1,26,30^ | - Identification and training of change leaders ^1,8,15,18,23,27,29,34,35,40^ - Role, responsibilities and expectations of change leaders ^12,14,23,40^ - Commitment and management from leadership to quality and safety ^3,8,9,25^ - Designated implementation coordinator ^5,37^ - Coordination and communication across disciplines ^5,34^ | - Supportive leadership for collaborative practice, interprofessional group of leaders, co-leading change ^1-3,7,8,11-19,22,23,28,31,35,37,38^ - Participative leadership ^2,7-9,12,14,22,23,26,27^ - Interprofessional feedback mechanisms, addressing the concerns, gaining consensus or trust ^1-3,8,12,15-17,19,23,24,30,31,34,35,37^ - Project’ prioritization by and commitment of the leaders, program coordinator, oversight, assistance ^1,3-5,7,9-11,13,16,19,22-27,30,31,34,38,39^ - Ongoing communication from leaders, highlighting expectations ^5,7,9,12,13,17,19,22,24,26,27,37-39^ - Recognized innovation champions (formal authority, role model, expertise, influence / inspiration, physical presence and proximity, persuasiveness, personal commitment, confidence, buy-in, information gathering, motivation) ^3,5-10,12-19,21-24,26-31,33,34,37,38^ - Multi-professional change agents / champions to lead the implementation, symmetry between champions ^8,12-14,16,22,28,33,34,37^ - Champions / leaders’ prior experience in project management ^8,12,26,33^ |
|  | Quality and fidelity monitoring / support | - Fragmentation of the project with little synergies, lack of workflow consistency or role clarity, monitoring difficulties ^12,20,31,37,39^ - Lack of project testing and monitoring, lack of referral, late identification of challenges ^5,10^ - Not considering the full change management process ^22^ - Lack of incentives to promote project adoption ^3^ | - Standards’ measure of adherence ^39^ - Measuring in routine care practice, assessing facility needs ^14,19,25,34^ - Stability in meeting external targets ^25^ - Consideration of enactment theory ^10^ - Coordination between daily business and implementation ^23^ | - Optimization of current professional practices / - enhancement of interprofessional collaborative practices ^1,2,5,10,12,16,20,22-24,27,30-32,34,35^ - Role formalization or clarification, legitimacy ^2,12,26^ - Breaking down the innovation in small / parallel projects /staging, planning ^16,22,24,32,34,36,39^ - Congruence with preexisting norms and beliefs ^1,4,34,36^ - Awards, MVP, internal competition, valorization, recognizing change efforts ^3,4,8,9,20,22,37^ - Use of guidelines or concept development, formal processes ^10,11,21-23,27,28^ - Process monitoring, audits, reporting, data collection, measuring compliance, sustaining change, routine evaluation ^1,5,9,10,12,14-20,22-25,27,28,31,32,34,35,37^ - Creating and sharing of artefacts, making change visible (stories, illustrations, poster, factsheets) ^22,27,28,32,36,37^ |
|  | Organizational staffing processes | - Turnover and understaffing, staff shortages of given professions ^1-3,5,8,22,23,28,32,33^ - Lack of specific background related to the innovation, insufficient training, poor onboarding ^2,3,5,11,12,26,32,33,38^ - Lack of recognition and consideration for professional or interprofessional background ^5,6,23,26,32^ - Inadequacy between project-related training and evolution of the project, time constraints for training ^24,30^ - Threat to relationships, interprofessional teamwork ^12^ | - Involvement in formal training relative to the innovation, professional and change management training ^1,2,11,12,18^ - Sufficient human resources ^4,8,10,25,29^ - Relationship building and teamwork ^8^ | - Availability of human resources, low staff turnover, onboarding process ^2,6,10,11,18,19,21,22,28,31^ - Increased availability or responsibility of given professions ^2,12,22,28,33,37^ - Specific knowledge / skills related to the innovation, literature search ^2,6,7,11,14,23,33^ - Formal / informal specialty oriented training linked with the project, continuous education ^1,6,8-12,14-16,20-24,27,28,30,32,35,37,38^ - Emphasis on interprofessional collaboration, vision sharing, identifying inter-professional challenges ^5,8,10-12,23,26,27,30,35^ |
|  | Individual characteristics | - Threat to professional or personal identity ^2,12,17,26^ - Departure of recognized influential change leaders ^2,23^ - Exhaustion, overwhelming and lack of enthusiasm, frustration ^2,3,7,8,10,12,14,23,26,31,32^ - Professions being “worked-around” ^23,32,39^ - Lack of identification with the innovation, resistance to change from specific professions / persons, lack of ownership ^1,3,4,7,12,14,17,22-24,26,27,29-34^ - Individual characteristics (lack of influence, non-volunteering) ^3,7,12^ | - Professional competence development, specific skills, educational benefits, perceptions ^2,3,6,7,9,12,29,37^ - Availability of early adopters, champions, personal characteristics ^8,25^ - Commitment and openness to engage in change, readiness assessment ^1,18^ | - Individual characteristics (influence, ownership, trust, motivation, voluntary, passion, competencies, team player) ^2,6-9,12,14,15,17,19,23-26,30,32,33^ - Openness to collaborative practice ^1,2,8,12,24,27,34^ - Commitment and passion of the champions, identification with the project, being convincing about the need for change ^1,4,8,10,13,17,21-23,26,27,30,33,34,37^ - Proactive dissemination and project advocacy by the users ^3,4,17,20-22,24-26,30^ - Finding resources in other, peer mutual support ^14^ - Professional satisfaction, empowerment ^12,15,21,24^ |

**References**

1. Antinaho T, Kivinen T, Turunen H, Partanen P. Increasing value-adding patient care by applying a modified TCAB program. *Leadership in health services (Bradford, England)*. Oct 2 2017;30(4):411-427. doi:10.1108/LHS-11-2016-0061

2. Barnett J, Vasileiou K, Djemil F, Brooks L, Young T. Understanding innovators' experiences of barriers and facilitators in implementation and diffusion of healthcare service innovations: a qualitative study. *BMC Health Serv Res*. Dec 16 2011;11:342. doi:10.1186/1472-6963-11-342

3. Hyzak KA, Bunger AC, Bogner JA, Davis AK. Identifying Barriers and Implementation Strategies to Inform TBI Screening Adoption in Behavioral Healthcare Settings. *J Head Trauma Rehabil*. Nov-Dec 01 2024;39(6):458-471. doi:10.1097/htr.0000000000001004

4. Engel M, van Zuylen L, van der Ark A, van der Heide A. Palliative care nurse champions' views on their role and impact: a qualitative interview study among hospital and home care nurses. Article. *BMC Palliat Care*. Feb 18 2021;20(1):34. doi:10.1186/s12904-021-00726-1

5. Vedel I, Le Berre M, Sourial N, Arsenault-Lapierre G, Bergman H, Lapointe L. Shedding light on conditions for the successful passive dissemination of recommendations in primary care: a mixed methods study. Article. *Implement Sci*. Oct 16 2018;13(1):129. doi:10.1186/s13012-018-0822-x

6. Adler-Milstein JR, Krueger GN, Rosenthal SW, Rogers SE, Lyles CR. Health system approaches and experiences implementing the 4Ms: Insights from 3 early adopter health systems. Article. *Journal of the American Geriatrics Society*. 2023;71(8):2627-2639. doi:10.1111/jgs.18417

7. Kitson A, Silverston H, Wiechula R, Zeitz K, Marcoionni D, Page T. Clinical nursing leaders', team members' and service managers' experiences of implementing evidence at a local level. *Journal of nursing management*. May 2011;19(4):542-55. doi:10.1111/j.1365-2834.2011.01258.x

8. Berger ER, Kreutzer L, Halverson A, et al. Evaluation of Changes in Quality Improvement Knowledge Following a Formal Educational Curriculum Within a Statewide Learning Collaborative. Article. *J Surg Educ*. Nov-Dec 2020;77(6):1534-1541. doi:10.1016/j.jsurg.2020.04.018

9. Yun L, Bilyk C, Bresson V, Brockmann J, Gordey L. Nurse champions as leaders for the implementation of CoACT Collaborative Care. Article. *Healthc Manage Forum*. May 2022;35(3):168-173. doi:10.1177/08404704221081993

10. Linke CA, Chapman LB, Berger LJ, Kelly TL, Korpela CA, Petty MG. Early Mobilization in the ICU: A Collaborative, Integrated Approach. *Crit Care Explor*. Apr 2020;2(4):e0090. doi:10.1097/CCE.0000000000000090

11. Flanagan ME, Plue L, Miller KK, et al. A qualitative study of clinical champions in context: Clinical champions across three levels of acute care. Article. *SAGE Open Med*. 2018;6:2050312118792426. doi:10.1177/2050312118792426

12. Bird M, McGillion M, Chambers EM, et al. A generative co-design framework for healthcare innovation: development and application of an end-user engagement framework. *Research involvement and engagement*. Mar 1 2021;7(1):12. doi:10.1186/s40900-021-00252-7

13. Johnson EE, Sterba KR, Goodwin AJ, et al. Implementation of an academic-to-community hospital intensive care unit quality improvement program: Qualitative analysis of multilevel facilitators and barriers. Article. *Annals of the American Thoracic Society*. 2019;16(7):877-885. doi:10.1513/AnnalsATS.201810-735OC

14. Shortell SM, Marsteller JA, Lin M, et al. The role of perceived team effectiveness in improving chronic illness care. Article. *Med Care*. Nov 2004;42(11):1040-8. doi:10.1097/00005650-200411000-00002

15. van de Baan FC, Lambregts S, Bergman E, Most J, Westra D. Involving Health Professionals in the Development of Quality and Safety Dashboards: Qualitative Study. *J Med Internet Res*. Jun 12 2023;25:e42649. doi:10.2196/42649

16. Abou Malham S, Breton M, Touati N, Maillet L, Duhoux A, Gaboury I. Changing nursing practice within primary health care innovations: the case of advanced access model. *BMC nursing*. Dec 2 2020;19(1):115. doi:10.1186/s12912-020-00504-z

17. Benn J, Burnett S, Parand A, Pinto A, Vincent C. Factors predicting change in hospital safety climate and capability in a multi-site patient safety collaborative: a longitudinal survey study. *BMJ Qual Saf*. Jul 2012;21(7):559-68. doi:10.1136/bmjqs-2011-000286

18. Laur C, Bell J, Valaitis R, Ray S, Keller H. The Sustain and Spread Framework: strategies for sustaining and spreading nutrition care improvements in acute care based on thematic analysis from the More-2-Eat study. Article. *BMC health services research*. 2018;18(1):930. doi:10.1186/s12913-018-3748-8

19. Lipshutz AK, Fee C, Schell H, et al. Strategies for success: A PDSA analysis of three QI initiatives in critical care. Article. *Jt Comm J Qual Patient Saf*. Aug 2008;34(8):435-44. doi:10.1016/s1553-7250(08)34054-9

20. Mayer CM, Cluff L, Lin WT, et al. Evaluating efforts to optimize TeamSTEPPS implementation in surgical and pediatric intensive care units. Article. *Jt Comm J Qual Patient Saf*. Aug 2011;37(8):365-74. doi:10.1016/s1553-7250(11)37047-x

21. Nowicki M, Berg BW, Okada Y, et al. A Patient Safety Champion Program for Interprofessional Health Care Educators: Implementation and Outcomes. *J Contin Educ Health Prof*. Jul 1 2022;42(3):211-218. doi:10.1097/CEH.0000000000000438

22. Olson CA, Tooman TR, Alvarado CJ. Knowledge systems, health care teams, and clinical practice: a study of successful change. Article. *Adv Health Sci Educ Theory Pract*. Oct 2010;15(4):491-516. doi:10.1007/s10459-009-9214-y

23. Riley M, Patterson V, Lane JC, Won KM, Ranalli L. The Adolescent Champion Model: Primary Care Becomes Adolescent-Centered via Targeted Quality Improvement. Article. *J Pediatr*. Feb 2018;193:229-236 e1. doi:10.1016/j.jpeds.2017.09.084

24. Hut-Mossel L, Ahaus K, Welker G, Gans R. Which Attributes of Credibility Matter for Quality Improvement Projects in Hospital Care-A Multiple Case Study among Hospitalists in Training. *Int J Environ Res Public Health*. Dec 6 2022;19(23)doi:10.3390/ijerph192316335

25. Gallagher K, Nutting PA, Nease DE, Jr., et al. It takes two: using coleaders to champion improvements in small primary care practices. *Journal of the American Board of Family Medicine : JABFM*. Sep-Oct 2010;23(5):632-9. doi:10.3122/jabfm.2010.05.090198

26. Basinska K, Wellens NIH, Simon M, Zeller A, Kressig RW, Zuniga F. Registered nurses in expanded roles improve care in nursing homes: Swiss perspective based on the modified Delphi method. *J Adv Nurs*. Feb 2021;77(2):742-754. doi:10.1111/jan.14644

27. Maynard GA, Budnitz TL, Nickel WK, et al. Mentored implementation: building leaders and achieving results through a collaborative improvement model. Innovation in patient safety and quality at the national level. Article. *Joint Commission journal on quality and patient safety / Joint Commission Resources*. 2012;38(7):301-310.

28. Borlaug G, Edmiston CE, Jr. Implementation of a Wisconsin Division of Public Health Surgical Site Infection Prevention Champion Initiative. Article. *AORN J*. May 2018;107(5):570-578. doi:10.1002/aorn.12123

29. Feldman SS, Schooley BL, Bhavsar GP. Health information exchange implementation: lessons learned and critical success factors from a case study. *JMIR Med Inform*. Aug 15 2014;2(2):e19. doi:10.2196/medinform.3455

30. Le Roux SR, Jassat W, Dickson L, et al. The role of emergent champions in policy implementation for decentralised drug-resistant tuberculosis care in South Africa. Article. *BMJ Glob Health*. Dec 2022;7(12)doi:10.1136/bmjgh-2022-008907

31. Rattray NA, Damush TM, Miech EJ, et al. Empowering Implementation Teams with a Learning Health System Approach: Leveraging Data to Improve Quality of Care for Transient Ischemic Attack. *J Gen Intern Med*. Nov 2020;35(Suppl 2):823-831. doi:10.1007/s11606-020-06160-y

32. Cherney RL, Pandian V, Ninan A, et al. The Trach Trail: A Systems-Based Pathway to Improve Quality of Tracheostomy Care and Interdisciplinary Collaboration. Article. *Otolaryngol Head Neck Surg*. Aug 2020;163(2):232-243. doi:10.1177/0194599820917427

33. Reuben DB, Evertson LC, Jackson-Stoeckle R, et al. Dissemination of a successful dementia care program: Lessons to facilitate spread of innovations. *Journal of the American Geriatrics Society*. Sep 2022;70(9):2686-2694. doi:10.1111/jgs.17900

34. Hohmeier KC, McKeirnan K, Akers J, et al. Implementing community pharmacy-based influenza point-of-care test-and-treat under collaborative practice agreement. *Implement Sci Commun*. Jul 16 2022;3(1):77. doi:10.1186/s43058-022-00324-z

35. Whitebird RR, Solberg LI, Jaeckels NA, et al. Effective implementation of collaborative care for depression: What is needed? Article. *American Journal of Managed Care*. 2014;20(9)

36. Hendy J, Barlow J. The role of the organizational champion in achieving health system change. *Social science & medicine (1982)*. Feb 2012;74(3):348-355. doi:10.1016/j.socscimed.2011.02.009

37. Doherty DP, Wood LJ, Durkin GJ. Strengthening Healthy Work Environment Outcomes Via Interprofessional Direct Care Champion Roles. Article. *J Nurs Adm*. Nov 1 2021;51(11):561-567. doi:10.1097/NNA.0000000000001064

38. Mello MM, Roche S, Greenberg Y, Folcarelli PH, Van Niel MB, Kachalia A. Ensuring successful implementation of communication-and-resolution programmes. *BMJ Qual Saf*. Nov 2020;29(11):895-904. doi:10.1136/bmjqs-2019-010296

39. Bonawitz K, Wetmore M, Heisler M, et al. Champions in context: which attributes matter for change efforts in healthcare? *Implement Sci*. Aug 6 2020;15(1):62. doi:10.1186/s13012-020-01024-9

40. Damschroder LJ, Banaszak-Holl J, Kowalski CP, Forman J, Saint S, Krein SL. The role of the champion in infection prevention: results from a multisite qualitative study. Article. *Quality & safety in health care*. Dec 2009;18(6):434-40. doi:10.1136/qshc.2009.034199

41. Hargraves D, White C, Frederick R, et al. Implementing SBIRT (Screening, Brief Intervention and Referral to Treatment) in primary care: lessons learned from a multi-practice evaluation portfolio. Review. *Public Health Rev*. 2017;38(1):31. doi:10.1186/s40985-017-0077-0
