# Supplementary material for: Implementing collaborative practices in healthcare settings using champions: a scoping review
Source: Implement Sci. 2025 Nov 4;20:48. doi: 10.1186/s13012-025-01463-2 (PMC12584293; doi:10.1186/s13012-025-01463-2)
Supplement: Supplementary file 5 — Supplementary Material 5. Appendix E Summary of recommendations highlighted with EPIS framework [file 13012_2025_1463_MOESM5_ESM.docx]

**Appendix E: Summary of recommendations highlighted with EPIS framework**

| Dimension | Construct | Recommendations |
| --- | --- | --- |
| Outer context | Service environment / policies | - Governmental policies, legislation, laws influencing innovation and implementation development ^1-8^ - National programs or organizations providing guidelines, quality labels or certifications ^1,3,4,7,9-20^ - Innovation’s consistency with current best practice models or EBP ^3,21,22^ - Insurance policies’ consistency with innovations’ implementation ^2,5,6,23^ - Favorable general context such as state of public health, rural / city environment, or infrastructures ^2,6,10,22,24,25^ |
|  | Funding / contracting | - Politic or institutional financial support for the innovation ^2-5,7,10,12,15,18-20,22,26-30^ - Favorable economic climate ^3^ - Suitability of insurance reimbursement terms with the innovation ^2,6,23,29^ |
|  | Leadership | - Top management support and commitment to innovation, top-down innovation’s promotion, exemplary value ^2,3,6-8,19,25,27,29,31,32^ - Availability of representation / representative for national program coordination, active participation and support from representative ^2,4,10,25,27,32^ - Process and standardization to ensure institutional functioning and collaboration, alignment, supra-ordinated objectives ^4,13,18,20^ |
|  | Inter-organizational environment and networks | - Inter- and intra-organizational sharing of innovation’s results, idea generation or mutualization, institutional coordination, participatory research ^3,4,6,7,10,11,14,15,17,22,29,33,34^ - Innovation dissemination through inter-organizational connections, bridging, innovation marketing ^3,4,10,13,17,18,27,29^ - Development of innovation-related partnerships: commitment, discussions, support, communication, trust, negotiations, network ^2-12,15,17,18,20,22,25,27,29,31,33-35^ |
|  | Patient / client characteristics | - Receptivity and integration of patients to the innovation: attractiveness, participation, interest, rewards ^1,6,14,22^ - Consideration of and response to patient characteristics in the innovation: patients’ condition, disease, multi-morbidity ^5,6,10,11,14,22,34,36^ |
|  | Patient / client advocacy | - Patients’ representative groups interest and commitment to the innovation, project promotion, dissemination ^3,6,15,18,22^ - Integration of patients’ representative groups in the innovation or its development ^15,18,36^ |
| Innovation factors | Innovation / EBP developers | - Possibility of prototyping, pilot project for the innovation ^4,13-15,28,31,37^ - Identification and presence of change agents such as early adopters, champions, inter-professional or –organizational change teams ^4,5,17,35-39^ - Experts’ adaptation to project development in the field, integration of field-representatives or spokespersons ^4,17,20,34,35^ - End-users consideration and integration, workflow analysis ^4,6,14,17,20,22,32,34-36,39^ - On-site presence of innovation’s developers, openness to innovation’s adaptation, on-site visits, teaching and data collection ^4,5,7,10,14,15,20,29,33^ |
|  | Innovation / EBP characteristics | - Suitability and compatibility of the innovation to the context ^1,4,6,8-10,12,17,18,28,29,31,33,36-38^ - Enhancement of professional work through change, dedicate time or means, gamification ^1,8,14,20,24^ - Innovation’s cost, existence of cheaper alternatives to the innovation, opportunity cost ^4,14,31^ - Innovation’s ease of understanding, implementation guides or training, innovation’s features ^2,4-6,8,13-16,18,20,23,28,29,31,33-35,38,40,41^ - Possibility to collect data and report, demonstration of the innovation’s impact, communication process ^3,5,8,14,17,24,28,29,33^ - Users involvement in innovation development, interprofessional project team ^4,5,14,17,25,29,31,36^ - Prioritization and fine-tuned planning of the implementation, step by step implementation or innovation’s modularity, availability of material or equipment, protocols ^4-7,10,12,15,17,18,20,28,33,36,38^ |
|  | Innovation / EBP fit | - Innovation’s adaptation to the context, implementation’s multi-modality, innovation’s integration into work routines, assessment of current practices, monitoring ^1,3-6,8,10,12-15,17,18,25,28-38,40^ - Consideration of expected tasks’ variability, known work standards ^1,4,5,8,14,30,33-35,40^ - Active role in the change process, end-users’ perceived meaning of the innovation, large-scale commitment ^1,3,6,17,25,28,32,33,35^ - Develop a community of practice, shared learning within a dedicated environment ^1,25,29^ - Proactive commitment with innovation’s partners to facilitate change, planning and training, contextual adjustments ^3,4,6,15,18,29,34,35,38^ - Congruency between institutional values, norms and beliefs and the innovation ^3,4,6,33^ |
| Bridging factors | Community-academic partnerships | - Data collection and publication of innovation’s results ^3,5^ - Integration of evidence (EBP, best practices) and recommendations within the innovation, literature search ^5,7,9,11,13-16,34,36^ - Familiarization with the local context, representativeness and proactive communication ^4,6,7,13,15,31,34,36^ - Close collaboration between research group and the field: co-design and marketing of the innovation or project ^4,7,10,11,14,17,18,20,22,25,34-36^ - Supervision and advice from domain experts, experience sharing, recognition of the need for change ^4,5,7,11,13,15-17,20,32,38^ |
|  | Purveyors / Intermediaries | - Presence of champions or clinical experts, interprofessional expert group, typology of the purveyors such as academics or quality / safety experts ^7-9,13,18,19,24,27-29,35,36,38,40^ - Preparation and training of internal champions, ongoing training and support, follow-up and monitoring processes ^6-8,13,16,20,22,24,34,35,37-39^ - External experts to assist implementation, support (administrative, logistical), commitment and understanding, collaboration, extra-institutional point of contact ^4,7,8,10,13,15,17,18,20,22,27,29,31,34-37,39^ |
| Inner context | Organizational characteristics | - Maximization of professional contributions, the right person in the right place, identification of key people ^1,2,4,12,13,30,38^ - Collegiality, cohesion and interprofessional connection ^1,6,8,11,12,15,21-23,25,27,31,37^ - Favorable institutional climate (culture, responsibility, support) and proactive communication ^1-4,6-8,11,13,16,21-23,25,26,30,32,38^ - Knowledge of the roles of other professions, interprofessional respect ^1,7,11,12,25,28,38^ - Availability of means/resources made for implementation, perceived support and backing ^2,4,6,7,14,15,19,21-23,25,27,30,33,40^ - Compatibility of innovation with other institutional projects or tasks, organizational priorities, workflow ^4-6,8,14,20,22,23,26,28^ - Characteristics of the institution, such as size, location, type, patient volume, organization, experience, network ^6,8,16,18,21-23,26,29-31,41^ |
|  | Leadership | - Autonomy in decision-making, resilience and flexibility, hierarchical level ^1,4,22-25,27^ - Supportive leadership of local managers, levers of change, mastery-expertise ^1-4,6-10,16,20-27,31,34,35,38,39^ - Leaders / managers that support participation (participative style) and collaboration / communication, project update ^1,4-8,10,13,17,20-25,28,31,32,35^ - Interprofessional team of managers supporting / following the project, interprofessional knowledge, need to integrate different professions into the project team, symmetry between members ^5,8,12,14,17-19,21,22,24,25,27-32,34,35,38,39^ - Management involvement in the implementation process, data collection, training, persuasion, motivation, empathy, role modeling, vision sharing ^2-4,6-13,17-21,23,25-27,29,31-33,35,38-40^ - Recognized project coordinator/internal champion, identification, formalized roles (expected characteristics), early involvement in the process, recognition by teams ^5,7,8,11-22,24,25,27-30,32-34,39^ |
|  | Quality and fidelity monitoring / support | - Clear definition of responsibilities and spheres of competence, identification by context, understanding of issues, validation ^1,4,5,8,9,11-14,17,20,27,33,36^ - Formalizing work processes and avoiding their fragmentation, interprofessionality, protocols, troubleshooting, new routines, vectors of change ^1,4-8,13-17,21,24,27-29,32-36,39,40^ - Promotion and measurement of project adherence, proximity and exchanges with the field, information, staff feedback ^5,7,13-15,17,24,26-29,36,40^ - Qualitative/quantitative monitoring of the implementation process, indicators - fidelity and validity, auditing, considering the entire change process ^4,5,7,8,10,12-15,17,18,20,21,24,26,28,31,33-36,39^ - Rewarding success, encouragement, competition (gamification) ^2,3,13-15,24^ - Continue implementation efforts over time after implementation ^14^ |
|  | Organizational staffing processes | - Having sufficient staff to ensure implementation and day-to-day work, dedicated staff/time, interprofessional relations ^1-5,7-10,13,14,21-26,28,30-32,39^ - Ongoing training in connection with large-scale innovations, project information, staff development, response to requests ^1,2,5-9,13,14,21,22,24,25,28,38,39^ - Continuing education on topics specific to medical and nursing specialties ^5,6,8,9,11,13-15,20,21,24,28,29,32,34,38^ - Training credits, recognition ^6,11,27,34,38^ - Development of continuous training blueprints, integration of new employees, inter-professional work, experience sharing and transfer to other departments ^4,5,7,9,13,14,16,21,31,38^ - Expertise of local champions in their own field of competence ^2,5,9,11,12,14,20,22-25,27,29,32^ |
|  | Individual characteristics | - Individual adjustments possible according to tasks, professions, sense of ownership ^1,4,7,13,23,25,27,29,32,33,35^ - Work or professional development, education and skills development, practical tools ^1-4,6,8,14,16,21,22,24,25,30,33,34,39^ - Clarifying uncertainties, building confidence, addressing concerns ^1,4,6-8,14,22,23,25,27,29,32,33,39^ - Retain / recruit key people in the implementation process, turnover anticipation ^4,10,22,25,26,32,40^ - Openness / acceptance / interest / attitudes towards people's innovations, being convinced of change, readiness, willingness to change ^3,4,6-8,12,13,16,18,21-27,29-33,37,39-41^ - Perseverance, enthusiasm, personal qualities, personal commitment, contagious passion, identification with the project, motivation ^2,6,8,11-13,19,21-25,27,32,33^ |

**References**

1. Abou Malham S, Breton M, Touati N, Maillet L, Duhoux A, Gaboury I. Changing nursing practice within primary health care innovations: the case of advanced access model. *BMC nursing*. Dec 2 2020;19(1):115. doi:10.1186/s12912-020-00504-z

2. Hyzak KA, Bunger AC, Bogner JA, Davis AK. Identifying Barriers and Implementation Strategies to Inform TBI Screening Adoption in Behavioral Healthcare Settings. *J Head Trauma Rehabil*. Nov-Dec 01 2024;39(6):458-471. doi:10.1097/htr.0000000000001004

3. Barnett J, Vasileiou K, Djemil F, Brooks L, Young T. Understanding innovators' experiences of barriers and facilitators in implementation and diffusion of healthcare service innovations: a qualitative study. *BMC Health Serv Res*. Dec 16 2011;11:342. doi:10.1186/1472-6963-11-342

4. Feldman SS, Schooley BL, Bhavsar GP. Health information exchange implementation: lessons learned and critical success factors from a case study. *JMIR Med Inform*. Aug 15 2014;2(2):e19. doi:10.2196/medinform.3455

5. Hargraves D, White C, Frederick R, et al. Implementing SBIRT (Screening, Brief Intervention and Referral to Treatment) in primary care: lessons learned from a multi-practice evaluation portfolio. Review. *Public Health Rev*. 2017;38(1):31. doi:10.1186/s40985-017-0077-0

6. Hohmeier KC, McKeirnan K, Akers J, et al. Implementing community pharmacy-based influenza point-of-care test-and-treat under collaborative practice agreement. *Implement Sci Commun*. Jul 16 2022;3(1):77. doi:10.1186/s43058-022-00324-z

7. Johnson EE, Sterba KR, Goodwin AJ, et al. Implementation of an academic-to-community hospital intensive care unit quality improvement program: Qualitative analysis of multilevel facilitators and barriers. Article. *Annals of the American Thoracic Society*. 2019;16(7):877-885. doi:10.1513/AnnalsATS.201810-735OC

8. Kitson A, Silverston H, Wiechula R, Zeitz K, Marcoionni D, Page T. Clinical nursing leaders', team members' and service managers' experiences of implementing evidence at a local level. *Journal of nursing management*. May 2011;19(4):542-55. doi:10.1111/j.1365-2834.2011.01258.x

9. Basinska K, Wellens NIH, Simon M, Zeller A, Kressig RW, Zuniga F. Registered nurses in expanded roles improve care in nursing homes: Swiss perspective based on the modified Delphi method. *J Adv Nurs*. Feb 2021;77(2):742-754. doi:10.1111/jan.14644

10. Borlaug G, Edmiston CE, Jr. Implementation of a Wisconsin Division of Public Health Surgical Site Infection Prevention Champion Initiative. Article. *AORN J*. May 2018;107(5):570-578. doi:10.1002/aorn.12123

11. Engel M, van Zuylen L, van der Ark A, van der Heide A. Palliative care nurse champions' views on their role and impact: a qualitative interview study among hospital and home care nurses. Article. *BMC Palliat Care*. Feb 18 2021;20(1):34. doi:10.1186/s12904-021-00726-1

12. Gallagher K, Nutting PA, Nease DE, Jr., et al. It takes two: using coleaders to champion improvements in small primary care practices. *Journal of the American Board of Family Medicine : JABFM*. Sep-Oct 2010;23(5):632-9. doi:10.3122/jabfm.2010.05.090198

13. Laur C, Bell J, Valaitis R, Ray S, Keller H. The Sustain and Spread Framework: strategies for sustaining and spreading nutrition care improvements in acute care based on thematic analysis from the More-2-Eat study. Article. *BMC health services research*. 2018;18(1):930. doi:10.1186/s12913-018-3748-8

14. Lipshutz AK, Fee C, Schell H, et al. Strategies for success: A PDSA analysis of three QI initiatives in critical care. Article. *Jt Comm J Qual Patient Saf*. Aug 2008;34(8):435-44. doi:10.1016/s1553-7250(08)34054-9

15. Maynard GA, Budnitz TL, Nickel WK, et al. Mentored implementation: building leaders and achieving results through a collaborative improvement model. Innovation in patient safety and quality at the national level. Article. *Joint Commission journal on quality and patient safety / Joint Commission Resources*. 2012;38(7):301-310.

16. Nowicki M, Berg BW, Okada Y, et al. A Patient Safety Champion Program for Interprofessional Health Care Educators: Implementation and Outcomes. *J Contin Educ Health Prof*. Jul 1 2022;42(3):211-218. doi:10.1097/CEH.0000000000000438

17. Olson CA, Tooman TR, Alvarado CJ. Knowledge systems, health care teams, and clinical practice: a study of successful change. Article. *Adv Health Sci Educ Theory Pract*. Oct 2010;15(4):491-516. doi:10.1007/s10459-009-9214-y

18. Reuben DB, Evertson LC, Jackson-Stoeckle R, et al. Dissemination of a successful dementia care program: Lessons to facilitate spread of innovations. *Journal of the American Geriatrics Society*. Sep 2022;70(9):2686-2694. doi:10.1111/jgs.17900

19. Whitebird RR, Solberg LI, Jaeckels NA, et al. Effective implementation of collaborative care for depression: What is needed? Article. *American Journal of Managed Care*. 2014;20(9)

20. Yun L, Bilyk C, Bresson V, Brockmann J, Gordey L. Nurse champions as leaders for the implementation of CoACT Collaborative Care. Article. *Healthc Manage Forum*. May 2022;35(3):168-173. doi:10.1177/08404704221081993

21. Flanagan ME, Plue L, Miller KK, et al. A qualitative study of clinical champions in context: Clinical champions across three levels of acute care. Article. *SAGE Open Med*. 2018;6:2050312118792426. doi:10.1177/2050312118792426

22. Vedel I, Le Berre M, Sourial N, Arsenault-Lapierre G, Bergman H, Lapointe L. Shedding light on conditions for the successful passive dissemination of recommendations in primary care: a mixed methods study. Article. *Implement Sci*. Oct 16 2018;13(1):129. doi:10.1186/s13012-018-0822-x

23. Bonawitz K, Wetmore M, Heisler M, et al. Champions in context: which attributes matter for change efforts in healthcare? *Implement Sci*. Aug 6 2020;15(1):62. doi:10.1186/s13012-020-01024-9

24. Doherty DP, Wood LJ, Durkin GJ. Strengthening Healthy Work Environment Outcomes Via Interprofessional Direct Care Champion Roles. Article. *J Nurs Adm*. Nov 1 2021;51(11):561-567. doi:10.1097/NNA.0000000000001064

25. Le Roux SR, Jassat W, Dickson L, et al. The role of emergent champions in policy implementation for decentralised drug-resistant tuberculosis care in South Africa. Article. *BMJ Glob Health*. Dec 2022;7(12)doi:10.1136/bmjgh-2022-008907

26. Benn J, Burnett S, Parand A, Pinto A, Vincent C. Factors predicting change in hospital safety climate and capability in a multi-site patient safety collaborative: a longitudinal survey study. *BMJ Qual Saf*. Jul 2012;21(7):559-68. doi:10.1136/bmjqs-2011-000286

27. Hendy J, Barlow J. The role of the organizational champion in achieving health system change. *Social science & medicine (1982)*. Feb 2012;74(3):348-355. doi:10.1016/j.socscimed.2011.02.009

28. Linke CA, Chapman LB, Berger LJ, Kelly TL, Korpela CA, Petty MG. Early Mobilization in the ICU: A Collaborative, Integrated Approach. *Crit Care Explor*. Apr 2020;2(4):e0090. doi:10.1097/CCE.0000000000000090

29. Mello MM, Roche S, Greenberg Y, Folcarelli PH, Van Niel MB, Kachalia A. Ensuring successful implementation of communication-and-resolution programmes. *BMJ Qual Saf*. Nov 2020;29(11):895-904. doi:10.1136/bmjqs-2019-010296

30. Shortell SM, Marsteller JA, Lin M, et al. The role of perceived team effectiveness in improving chronic illness care. Article. *Med Care*. Nov 2004;42(11):1040-8. doi:10.1097/00005650-200411000-00002

31. Damschroder LJ, Banaszak-Holl J, Kowalski CP, Forman J, Saint S, Krein SL. The role of the champion in infection prevention: results from a multisite qualitative study. Article. *Quality & safety in health care*. Dec 2009;18(6):434-40. doi:10.1136/qshc.2009.034199

32. Hut-Mossel L, Ahaus K, Welker G, Gans R. Which Attributes of Credibility Matter for Quality Improvement Projects in Hospital Care-A Multiple Case Study among Hospitalists in Training. *Int J Environ Res Public Health*. Dec 6 2022;19(23)doi:10.3390/ijerph192316335

33. Rattray NA, Damush TM, Miech EJ, et al. Empowering Implementation Teams with a Learning Health System Approach: Leveraging Data to Improve Quality of Care for Transient Ischemic Attack. *J Gen Intern Med*. Nov 2020;35(Suppl 2):823-831. doi:10.1007/s11606-020-06160-y

34. Riley M, Patterson V, Lane JC, Won KM, Ranalli L. The Adolescent Champion Model: Primary Care Becomes Adolescent-Centered via Targeted Quality Improvement. Article. *J Pediatr*. Feb 2018;193:229-236 e1. doi:10.1016/j.jpeds.2017.09.084

35. van de Baan FC, Lambregts S, Bergman E, Most J, Westra D. Involving Health Professionals in the Development of Quality and Safety Dashboards: Qualitative Study. *J Med Internet Res*. Jun 12 2023;25:e42649. doi:10.2196/42649

36. Bird M, McGillion M, Chambers EM, et al. A generative co-design framework for healthcare innovation: development and application of an end-user engagement framework. *Research involvement and engagement*. Mar 1 2021;7(1):12. doi:10.1186/s40900-021-00252-7

37. Antinaho T, Kivinen T, Turunen H, Partanen P. Increasing value-adding patient care by applying a modified TCAB program. *Leadership in health services (Bradford, England)*. Oct 2 2017;30(4):411-427. doi:10.1108/LHS-11-2016-0061

38. Cherney RL, Pandian V, Ninan A, et al. The Trach Trail: A Systems-Based Pathway to Improve Quality of Tracheostomy Care and Interdisciplinary Collaboration. Article. *Otolaryngol Head Neck Surg*. Aug 2020;163(2):232-243. doi:10.1177/0194599820917427

39. Mayer CM, Cluff L, Lin WT, et al. Evaluating efforts to optimize TeamSTEPPS implementation in surgical and pediatric intensive care units. Article. *Jt Comm J Qual Patient Saf*. Aug 2011;37(8):365-74. doi:10.1016/s1553-7250(11)37047-x

40. Adler-Milstein JR, Krueger GN, Rosenthal SW, Rogers SE, Lyles CR. Health system approaches and experiences implementing the 4Ms: Insights from 3 early adopter health systems. Article. *Journal of the American Geriatrics Society*. 2023;71(8):2627-2639. doi:10.1111/jgs.18417

41. Berger ER, Kreutzer L, Halverson A, et al. Evaluation of Changes in Quality Improvement Knowledge Following a Formal Educational Curriculum Within a Statewide Learning Collaborative. Article. *J Surg Educ*. Nov-Dec 2020;77(6):1534-1541. doi:10.1016/j.jsurg.2020.04.018
